# Case of Disunited Fracture Successfully Treated with the Seton

**Published:** 1841-08

**Authors:** William H. Donne

**Affiliations:** Louisville, Ky.


Art. II.—*Case of Disunited Fracture successfully treated with the Seton.* By William H. Donne, M. D. Reported by A. Martin, resident student in the Louisville Marine Hospital.

William Wheeling, a laborer, a native of Ireland, aged 23 years, of intemperate habits, was admitted into the Louisville Marine Hospital on the 14th June, 1840, with a transverse fracture of the tibia about three inches below the femoro-tibial articulation, caused by falling into the hold of a steamboat and striking the limb against a bar of iron. There was no apparent displacement of the fragments, and very little tumefaction of the soft parts was observed in the vicinity of the fracture. On moving the limb in various directions, crepitation was distinctly audible, and the superior fragment could be made slightly prominent by flexing the knee. No fracture of the fibula, however, could be detected upon the most careful examination. The roller was firmly applied by Dr. T. L. Caldwell, then in attendance, from the toes upwards above the point of fracture, with injunctions to moisten it as often as necessary with aqua ammoniæ and diluted alcohol. After the tumefaction had subsided, two splints, composed of book-binders' board, previously soaked in warm water, were applied and moulded to the limb by the roller, which was carried above the point of fracture as in the first dressing. The limb was now placed on an inclined plane, with directions to keep the dressings immediately over the fracture moistened from time to time. This treatment was continued for six weeks, when the splints, &c. were removed. Upon examination it was ascertained that no union had taken place. All dressings were then discontinued, and the patient permitted to take exercise on crutches through the ward, for the space of three weeks without any improvement. Friction, and rubbing the fragments together by moving the limb in different directions, were likewise resorted to without any beneficial results. On the 21st of August, with the concurrence and assistance of Dr. T. L. Caldwell, (whose tour of attendance had expired a few weeks previously,) Dr. Donne introduced a seton between the fragments, by first dividing the integuments and aponeuroses in the anterior tibial region to a small extent. The tibialis anticus muscle presenting itself, a few of its fibres were detached from their connection with the outer surface of the tibia, to facilitate the passage of the needle. The hemorrhage was comparatively slight, nor did the patient complain of much pain during the operation.

Five days elapsed before there was any appearance of inflammation, which increased gradually until the 27th, when the wound made by the egress of the needle began to suppurate.

29th. Suppuration in both wounds; patient complains of a stinging pain in the vicinity of the fracture.

31st. Pain more severe; inflammation much diffused.

Sept. 2nd. Complains of great pain; local inflammation increased.

4th. Scultetus' bandage applied, moistened with ammoniated lotion.

7th. Inflammation still extending; some febrile action; tongue furred; restless through the night; bowels open without medicine. Seton removed; a considerable quantity of cream coloured pus with some coagula discharged from the internal aperture.

10th. Internal wound continues to discharge healthy matter.

12th. The quantity of matter diminished; a compress with an aperture in its centre, and Scultetus' bandage applied.

14th. Discharge much diminished; roller applied.

17th. No pain in the limb; use splints and roller.

20th. Wounds closed: ordered porter and generous diet.

25th. Severe pain in the fractured limb; restless during the night.

26th. Bandages and splints removed; inflammation of the integuments and subcutaneous cellular tissue in the vicinity of the internal wound; limb much swollen; fluctuation evident; about eight ounces of sanguineo purulent matter discharged.

27th. Matter again collected; counter opening made—affording a free discharge.

Oct. 1st. Discharge very slight; pain greatly diminished by incision.

4th. Health improving; discharge ceased; counter opening closed.

8th. Free from pain; limb seems firm; no appreciable motion in the site of the fracture.

12th. Tumefaction of the limb diminished; patient exercises on crutches, and can support more weight on the leg than at any previous time since the accident.

16th. Patient walks about the ward with a cane; has a good appetite, and rests well at night.

20th. Bandages dispensed with; limb free from pain and daily gaining strength.

28th. Patient can bear his whole weight on the limb; walks well without any inconvenience.

A few weeks afterwards he was discharged cured.

There seems to be much discrepancy of opinion relative to the length of time that the seton should be retained in cases of disunited fracture; Dr. Gibson and others insisting on its retention for months, or until the deposition of ossific matter shall have commenced, while Mr. Liston, who is equally orthodox, thinks one or two weeks sufficient.

Nothing definite, we imagine, can be laid down as to the time which may be required to induce ossific deposition by the seton, for not only are constitutional peculiarities to be taken into consideration, but the location of the injury, and many adventitious circumstances, must influence the recuperative efforts of nature.

